# Metabolomic effects of the colonization of *Medicago truncatula* by the facultative endophyte *Arthrobacter agilis* UMCV2 in a foliar inoculation system

**DOI:** 10.1038/s41598-020-65314-4

**Published:** 2020-05-21

**Authors:** Arturo Ramírez-Ordorica, Eduardo Valencia-Cantero, Idolina Flores-Cortez, María Teresa Carrillo-Rayas, Ma. Isabel Cristina Elizarraraz-Anaya, Josaphat Montero-Vargas, Robert Winkler, Lourdes Macías-Rodríguez

**Affiliations:** 1 Instituto de Investigaciones Químico Biológicas, Universidad Michoacana de San Nicolás de Hidalgo, Edifico B3, Ciudad Universitaria, C. P. 58030 Morelia, Michoacán México; 2 Department of Biotechnology and Biochemistry, Cinvestav Unidad Irapuato. Km. 9.6 Libramiento Norte Carr. Irapuato-León. C. P. 36824, Irapuato, Guanajuato México

**Keywords:** Plant symbiosis, Microbial ecology

## Abstract

Biofertilizer production and application for sustainable agriculture is already a reality. The methods for biofertilizers delivery in crop fields are diverse. Although foliar spray is gaining wide acceptance, little is known about the influence that the biochemical features of leaves have on the microbial colonization. *Arthrobacter agilis* UMCV2 is a rhizospheric and endophytic bacteria that promotes plant growth and health. In this study, we determined the capacity of the UMCV2 strain to colonize different leaves from *Medicago truncatula* in a foliar inoculation system. By using two powerful analytical methods based on mass spectrometry, we determined the chemical profile of the leaves in 15-d old plants. The metabolic signatures between the unifoliate leaf (m1) and the metameric units developing above (m2 and m3) were different, and interestingly, the highest colony forming units (CFU) was found in m1. The occurrence of the endophyte strongly affects the sugar composition in m1 and m2 leaves. Our results suggest that *A. agilis* UMCV2 colonize the leaves under a foliar inoculation system independently of the phenological age of the leaf and it is capable of modulating the carbohydrate metabolism without affecting the rest of the metabolome.

## Introduction

Plants live in a close relationship with different microbial communities that reside inside or outside the plant tissues. The inside colonizers are referred as endophytes (from the Greek *endon*-within and *phyton*-plant), and when they are inside the plant, they completely depend on the plant and its internal conditions for growth^1–5^. A study of *Arabidopsis thaliana* grown under natural conditions showed that the bacterial community associated with the outside of the roots were more diverse than in the endophytic community, while the opposite pattern was observed in the leaves^6^. In addition, the authors found that leaves and roots shared many species of bacterial endophytes, suggesting that some root endosphere colonizers migrate and are capable of colonizing above-ground tissues; thus the microbial traits for competition and colonization influence the richness and composition of the plant endophytes^7,8^. According to the literature, the endophytes are non-pathogenic, but some of them could act as latent pathogens that can cause disease, depending on the stressful environmental circumstances^4,9^. Studies of the functional capacity of beneficial endophytes have revealed their potential as promoters of plant growth and health, moreover, they are free from the environmental pressure of changing soil and climate conditions experienced by their rhizospheric counterparts, so they are an excellent candidate for biofertilizers^10,11^. The delivery methods of endophytes in agriculture include seed inoculation, soil drenching, stem injection, and foliar fertilization. Currently, there is a renewed interest in a foliar-treated plants and different studies on the effect of bacterial and fungal endophytes on the growth, crop production, nutrient uptake, and defense have appeared in the literature^12–14^.

During the endophyte’s colonization, different molecular signaling events occur in the host plant^9,15^. With the development of more powerful analytical methods, many of the molecules involved in the colonization process have been identified. Untargeted methods based on mass spectrometry, such as direct liquid injection-electrospray ionization-mass spectrometry (DLI-ESI-MS) have increased in popularity in recent years. DLI-ESI-MS shows the broad molecular weight range of compounds present in samples and has been used successfully for metabolomics screening in plants during their interaction with microbes^16^ or for studying biochemical changes due to physiological state or in response to the environment^17–19^.

*Arthrobacter agilis* UMCV2 is a salmon-pigmented rhizospheric and endophytic actinobacterium that systematically colonizes the leaves of its host^20^. The bacterium has great potential as a biofertilizer, since it is capable of improving the nutritional status of plants by promoting iron acquisition processes that involve the reduction and dissolution of Fe^3+^ present in the soil^21–23^. In addition, analyses made by gas chromatography in tandem with mass spectrometry (GC-MS) indicated that the UMCV2 strain interacts with leguminous and monocot plants through the production of volatile compounds, promoting their growth and health^16,21–24^. A study using the DLI-ESI-MS method showed that volatiles from *A. agilis* UMCV2 induce the accumulation of different metabolites involved in the iron-adaptive processes in *Medicago truncatula*^16^.

Foliar inoculation could be a delivery method for introducing *A. agilis* UMCV2 into fields in order to enhance the plant growth and crop production, furthermore, it is timely to conduct a metabolomic approach to determine whether the interconnected leaf phenological age and chemical composition influence the growth of the endophytes in the leaves, and at the same time to gain insight into plant response to the endophytic colonization.

## Results

### Chemical signature of *M. truncatula* leaves

**Figure 1:**
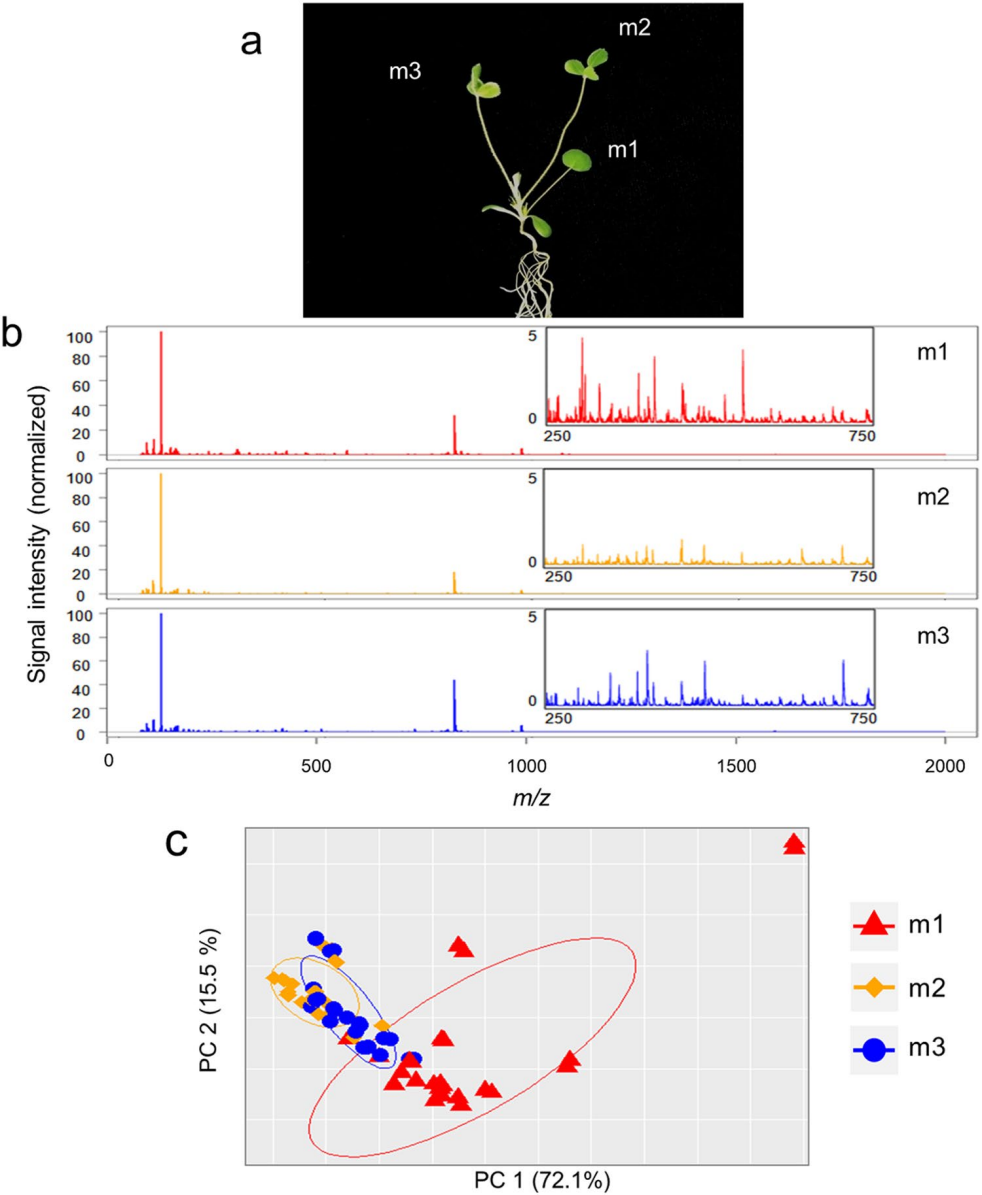
Metabolic phenotyping of *Medicago truncatula* leaves by DLI-ESI-MS. (**a**) Representative photograph of a 15-day-old *M. truncatula* plant, illustrating the numerical nomenclature used to name the three leaves as m1, m2, and m3. (**b**) Mass spectra from control m1, m2, and m3 leaves. Inset: expanded view of each mass spectra region at 250 to 750 *m/z*. (**c**) Principal component analysis of the metabolites obtained from DLI-ESI-MS in *M. truncatula* m1, m2, and m3 leaves (n = 24 for each leaf). PERMANOVA (α = 0.05).

**Figure 2:**
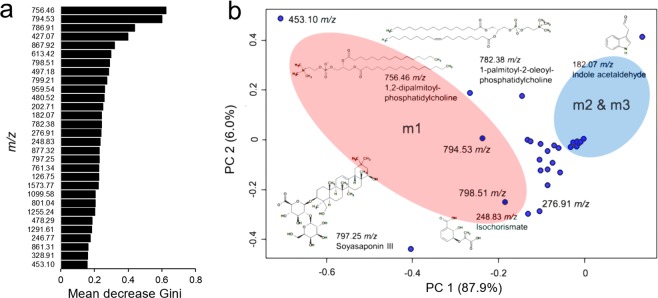
Ranking of ion importance using the Random Forest model (**a**) and the loading plot of the principal component analysis (PCA) results (**b**) for discrimination of the phenological age of *M. truncatula* leaves with the DLI-ESI-MS data. Colored ovals in the PCA represent a confidence interval of 95%. Some relevant ions were putatively identified using SpiderMass software and the PlantCyc database.

Control leaves were characterized using metabolic phenotyping by DLI-ESI-MS. This tool provided the broad molecular weight range of compounds in the leaves. The mass spectra obtained showed 1121 ions, but the ion intensities in the unifoliate leaf (m1) were different from those obtained in the first (m2) and second trifoliate leaves (m3) (Fig. 1a,b). The ordering analysis clearly separated the m1 from the m2 and m3 samples with a high significance (*p* < 0.001), contrasted using PERMANOVA (Fig. 1c). To determine the most important ions that distinguish m1 from m2 and m3 leaves, we constructed a Random Forest (RF) model that delivered the 30 most important ions (Fig. 2a). The PCA from the selected ions displayed a group belonging to m1 with an error of less than 1% (Fig. 2b), thus, the separation of m1 from m2 and m3 was even greater after the ion selection (Figs. 1c and 2b). In addition, the RF model did not distinguish between m2 and m3, with an error of up to 40%. These results suggest that the physiological condition of the m1 leaf has a clearly distinct metabolic identity compared to the m2 and m3 leaves, which presented the same chemical profile at the time of harvest.

The DLI-ESI-MS results of the 30 ions were compared with the SpiderMass database. Only 13 ions were putatively identified (Table 1). Precursors of phytohormones (182.07, 427. 07, 497.18 *m/z*), protochlorophyllide (613.42 *m/z*), defense-related compounds (248.83, 478.29 *m/z*), structural components of cell membranes (756.46, 782.38, 786.91 *m/z*), and a soyasaponin (797.25 *m/z*), were positively identified. The remaining ions did not match any compounds in the database and were classified as unknown (Table 1).

**Table 1:** Putative identification of the main ions obtained by DLI-ESI-MS analyses and SpiderMass software.

*m/z* (±0.35)	Monoisotopic Mass [Da]	Ionization mode	Common name	Description
182.07	159.06842	[M + Na]+	Indoleacetaldehyde	Indolic, auxin-related compound. Precursor of indole-3-acetic acid in the indole pyruvate route^44–46^
248.83	226.047745	[M + Na]+	Isochorismate	Intermediary in the biosynthesis of salicylic acid^38–41^
427.07	426.386169	[M + H] +	Amyrin	Intermediary in the synthesis of triterpenes^71^
478.29	455.353058	[M + Na]+	Oleanolate	Intermediary in the synthesis of triterpenes and with proven antimicrobial activity^71^
497.18	496.339996	[M + H] +	26-Hydroxybrassinolide	Brassinosteroid inactivated form^43^
613.42	612.22229	[M + H] +	Protochlorophyllide a	Intermediary in the biosynthesis of chlorophyll^36^
756.46	733.562134	[M + Na]+	1,2-Dipalmitoyl-phosphatidylcholine	Cell membrane phospholipid^36,37^
782.38	759.57782	[M + Na]+	1-Palmitoyl-2-oleoyl-phosphatidylcholine	Cell membrane phospholipid^36,37^
786.91	785.593445	[M + H] +	1-Oleoyl-2-oleoyl-phosphatidylcholine	Cell membrane phospholipid^36,37^
797.25	796.460938	[M + H] +	Soyasaponin III	Saponin with defense effect^45,71^
867.92	867.131226	[M + H] +	Methylmalonyl-CoA	Product of degradation of branched amino acids, possibly implicated in the biosynthesis of waxes^72^
877.32	854.285767	[M + Na]+	Preuroporphyrinogen	Intermediary in the biosynthesis of chlorophyll^36^
959.54	936.580994	[M + Na]+	1,2-Dilinolenoyl-digalactosyldiacylglycerol	Thylakoid membrane component^36,37^
Unknown ions (*m/z*): 126.75, 202.71, 246.77, 276.91, 328.91, 453.09, 480.52, 761.34, 794.53, 798.51, 799.21, 801.04, 861.31, 1099.58, 1255.24, 1291.61, 1573.42				

Tolerance ± 0.35.

The relative signal magnitude of the 182.07 *m/z* ion that matched to indole acetaldehyde in the database was higher in m2 and m3 leaves, whereas the magnitudes of the remaining ions were all higher in m1 leaves. Thus, the 182.07 *m/z* ion could be established as a metabolite marker for the phenological age of *M. truncatula* leaves (Fig. 2b).

### Detection and quantification of *A. agilis* UMCV2 in internal leaf tissues

**Figure 3:**
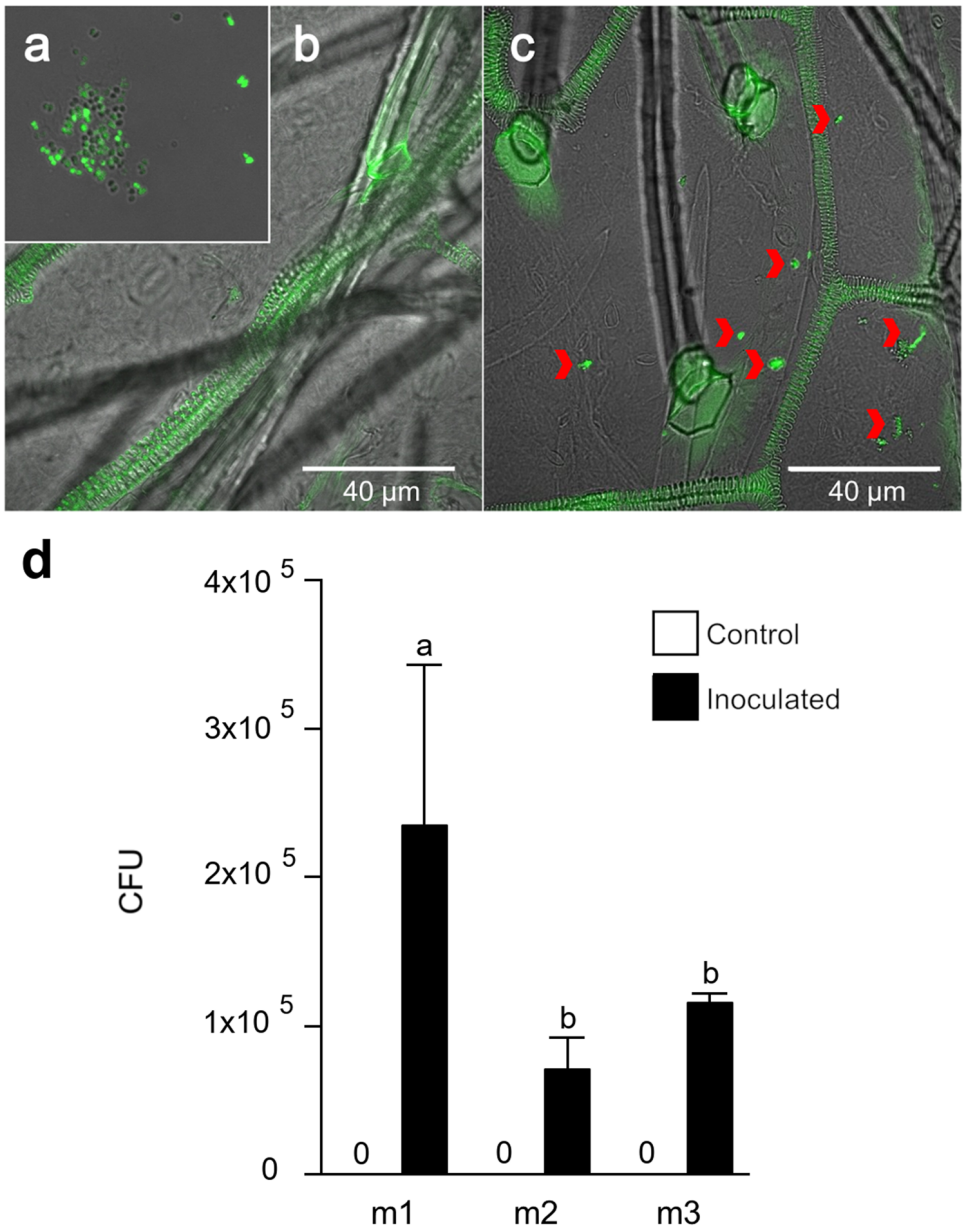
*Arthrobacter agilis* UMCV2 colonization in *Medicago truncatula* leaves following foliar inoculation. (**a**) Fluorescence *in situ* hybridization assay of the pure microbial culture. (**b**) Representative photograph of the control and inoculated m1 leaf (**c**). Arrowheads indicate the presence of microcolonies within the tissue. (**d**) Quantification of *A. agilis* UMCV2 in the leaf tissues 5 days after foliar inoculation. Bars represent the means ± standard error values (n = 8). The same letter represents no significant differences between means by Tukey’s post-test (α = 0.05).

The anatomical structure and chemical features of leaves are presumed to affect the internalization, survival, and growth of the endophytes^7,25^. In regards to the chemical landscape from different metameric units of *M. truncatula*, we determined that the chemical profile of the m1 leaf was dissimilar to those of m2 and m3; nevertheless, this difference might not influence the endophytic colonization of *A. agilis* UMCV2. The combination of FISH (Fig. 3a–c), qPCR technique, and total viable account determination (Fig. 3d) showed that the endophyte is able to colonize the m1, m2, and m3 leaves after foliar inoculation. Furthermore, m1 showed the highest colonization, while m2 and m3 showed no differences in bacterial DNA content (*p* = 0.1687) (Fig. 3d). In addition, we measured the bacterial population sizes in m1 leaves at different post-inoculation times (24, 72, and 120 h); again, no significant differences were observed in the CFUs. The inoculation experiment was repeated with the half of the cells in the inoculum but no significant differences were observed in this experiment either (Supplementary Fig. S1), showing that the size of the initial inoculation concentration did not modify the final colonization obtained, tending to stabilize to values lower than 1 × 10^6^ CFU/leaf.

### Metabolite profiling and sugar content in leaves during plant-microbe interaction

**Figure 4:**
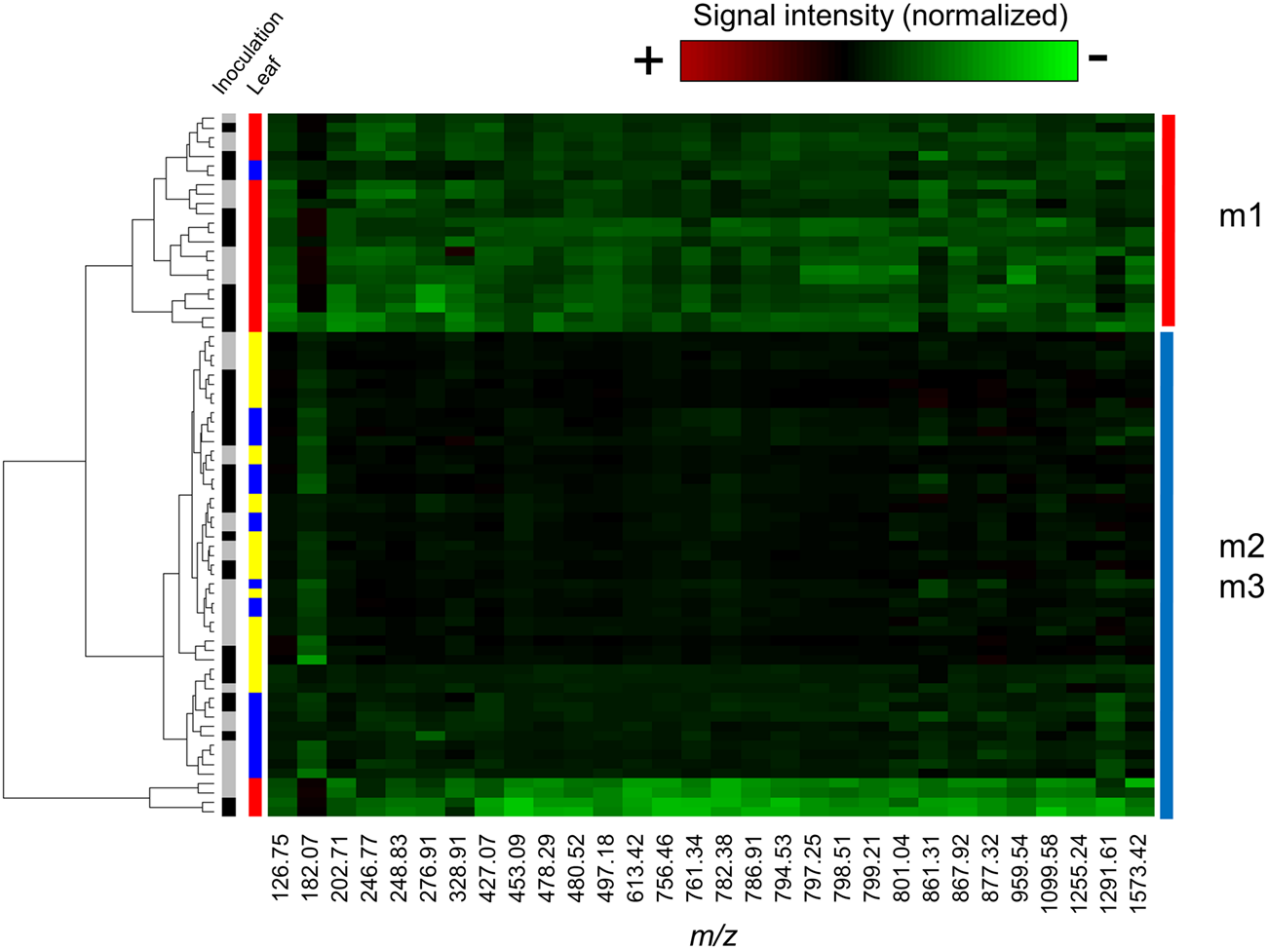
Metabolic heat map of the ion signal intensities selected by the Random Forest model in control and inoculated leaves of *Medicago truncatula*. The red, yellow, and blue marks correspond to m1, m2, and m3 leaves, respectively. The black and grey marks correspond to control and inoculated leaves, respectively. The control m1 leaf clearly clusters outside the m2 and m3 leaves. No differences were found between control and inoculated leaves. The heat map demonstrates the high repeatability of the measurements.

To determine if the metabolite profile changed after the inoculation, we analyzed the leaves by DLI-ESI-MS at day 5 of the interaction. As the ions obtained were the same for both the inoculated and uninoculated leaves, a heat map was constructed with the ions selected by the RF model (Fig. 4). The hierarchical clusters grouped the samples according to the leaf development and inoculation. The control m1 leaf samples clustered separately from the m2 and m3 samples, which were indistinguishable. Furthermore, it was not possible to associate the global chemical changes obtained by DLI-ESI-MS with the occurrence of the endophyte. As DLI-ESI-MS did not cover the complete metabolome, we performed a GC-MS analysis as a complementary method for the identification and quantification of monosaccharides and disaccharides in the leaves. A factorial analysis performed with the results obtained by the chromatographic determinations showed differences in sugar content between the control and inoculated leaves (*p* = 1.12 × 10^−5^) (Table 2). Fructose and ethyl-D-glucopyranoside accumulated more in inoculated m1 leaves compared to uninoculated controls; in contrast, glucose and myo-inositol were more abundantly detected in inoculated m2 leaves. Sucrose also was identified, but no differences in levels were found between treatments. Furthermore, no statistical differences in individual or total sugar content (glucose, fructose, myo-inositol, sucrose, and ethyl-D-glucopyranoside) were found between control and inoculated m3 leaves. However, total sugars showed the highest values in the m1 leaves, which corresponded to the largest bacterial colonization.

**Table 2:** Sugar content in control and inoculated leaves of *Medicago truncatula*.

Sugars (µg/g FW)	Control			*A. agilis* UMCV2		
	m1	m2	m3	m1	m2	m3
D-Glucose	8.11 ± 1.19 b	9.53 ± 0.94 b	10.61 ± 0.52 ab	12.22 ± 0.39 ab	17.33 ± 3.34 a	9.67 ± 1.64 b
D-Fructose	192.96 ± 18.72 b	96.10 ± 12.29 b	180.87 ± 25.40 b	407.66 ± 107.99 a	158.04 ± 9.99 b	115.56 ± 21.66 b
Myo-inositol	169.85 ± 7.00 ab	103.57 ± 21.39 b	118.13 ± 13.13 b	171.50 ± 6.01 ab	209.42 ± 9.95 a	78.61 ± 22.79 b
Ethyl-D-glycopiranoside	661.08 ± 81.23 b	545.18 ± 15.30 bc	437.47 ± 15.61 c	927.90 ± 32.10 a	716.59 ± 47.98 b	415.78 ± 17.69 c
Sucrose	31.50 ± 2.21 a	33.10 ± 3.30 a	25.50 ± 0.91 a	79.47 ± 28.03 a	50.06 ± 25.11 a	26.01 ± 1.09 a
Total	1055.38 ± 72.42 b	771.33 ± 26.14 c	755.56 ± 35.60 c	1586.53 ± 60.83 a	1124.11 ± 63.59 b	625.55 ± 51.73 c

Data shown are means ± SE for samples from 7 replicates. Control leaves were collected from 15-day-old plants. Plants were inoculated at 10 days of age, and the interaction lasted for 5 days. The same letter represents no significant differences between means by a two factors ANOVA followed by a Tukey´s post-test (*p* ≤ 0.05).

## Discussion

Plant growth promoting rhizobacteria (PGPR) are microorganisms that greatly influence plant growth and productivity and exert an optimal effect on plant health^26^; therefore, in crop fields, they are either irrigated in the rhizosphere or applied as foliar spray^13,27,28^. Some PGPR can colonize plants and thrive inside the tissues, gaining access to the host plants via openings in the root or through stomata, wounds, and hydathodes in the shoot when they fall on the foliage either by artificial inoculation or because of the wind^11^.

The rhizobacterium *A. agilis* UMCV2 used in this study is a facultative endophyte, and has been shown to promote the growth and development of *M. truncatula* under iron-sufficient and -deficient growth conditions^21,22,29,30^. This study was performed to determine if *A. agilis* UMCV2 was able to colonize the different metameric units of *M. truncatula* seedlings under a foliar inoculation system. In addition, taking advantage of this form of bacterial inoculation, we performed a metabolomic profiling to determine whether the chemical composition linked to the phenological age of the leaves influences endophyte growth and colonization. First, we analyzed the metabolite profile of *M. truncatula* leaves to discern the chemical differences between them. At the time of the harvest, the seedlings had one unifoliate leaf (m1), and two trifoliate leaves (m2 and m3). Using DLI-ESI-MS and statistical models for data analysis, we determined that the metabolite profile of m1 was different from that of m2 or m3. Although the ion signals were the same, the intensities of many of them displayed higher relative abundances in m1 leaves. The m1 leaf is the first true leaf produced by the epicotyl; the meristematic region of the epicotyl then continues to grow, adding trifoliate leaves on alternate sides of the developing stem^31^. Thus, the differences in ion intensities among the leaves may correspond to the leaf phenological age^32–35^. Some of the compounds putatively identified are part of the membranes (e.g., 1, 2-dilinolenoyl-digalactosyldiacylglycerol). Monogalactosyldiacylglycerol (MGDG) and digalactosyldiacylglycerol (DGDG) constitute 80% of the total membrane lipids of the thylakoid membranes and they act directly in several important plastid roles, particularly during photosynthesis^36,37^. In contrast, isochorismate is involved in plant defense responses as a precursor of salicylic acid (SA), a phenolic phytohormone widely studied for its role in activating defense responses during phytopathogen attacks^38^. Indeed, SA accumulation in older leaves has been positively correlated with increased disease resistance compared to the youngest leaves^39–41^. Similarly, saponins are also involved in plant defenses against herbivores and pathogens, in addition to having roles as allelopathic agents^42^.

Brassinosteroids are steroids that regulate many aspects of plant growth and development, especially in stress adaptation^16^. They act both independently and in conjunction with other phytohormones, controlling different plant metabolic activities. The brassinosteroid that we found in the m1 leaf is hydroxylated at C-26, leading the inactive form;^43^ it should be noted that the most bioactive form named brassinolide was previously identified in *M. truncatula* by using a targeted analysis by GC-MS^16^. Additionally, we found increased levels of the auxin-related compound indole-3-acetaldehyde (182.07 *m/z*) in the youngest leaves (m2 and m3). This compound acts as storage compound for the production of indole-3-acetic acid^44^. Auxins induce G1 to S (G1/S) phase transition of the cell cycle, promoting proliferation, cell wall laxity, and cell expansion^45,46^. Thus, the presence of indole-3-acetaldehyde in m2 and m3 leaves is a biomarker of the leaf phenological age in *M. truncatula* seedlings.

With a complementary study using GC-MS, we monitored quantitative differences in soluble sugar content in the leaves (e.g., D-glucose, D-fructose, myo-inositol, ethyl D-glucopyranoside, and sucrose). These small sugars are critical for plant growth, development, and stress responses^47,48^. Measurement of the total sugar concentration in each leaf indicated that m1 leaves accumulate more sugars than m2 and m3. According to the literature, the unifoliate leaf is a source tissue that supports the growth and development of sink tissues displaying active growth through sucrose translocation^49^.

At 5-d of foliar inoculation and using a confocal microscope, we observed bacterial microcolonies in the m1, m2, and m3 leaves, indicating that *A. agilis* UMCV2 is able to internalize and colonize all the tissues independently of the physicochemical features of the leaves. The pattern of colonization followed by endophytes is typically not homogenous and it is specific of the microbial taxon and strain type and also they present affinity towards their hosts^25,50–52^. Therefore, physiological parameters such as plant genotype, developmental stage, nutritional status, type of organs (leaf vs. root), leaf age, among others, are involved in the colonization process^6,7,10^. The present study shows that *A. agilis* UMCV2 colonizes the leaves of *M. truncatula*, and the plant maintains the colonization independently of the metabolic differences between them. A heavy inoculation does not produces significate differences in the colonization of m1 leaves over time. Previous studies have indicated that endophytes must pass the first line of defense of the plant immune system and determine how to protect themselves from it, but at the same time, the plants have different defense strategies to keep microbial growth in control^53,54^. Thus, an exchange of numerous chemical signals between the plant and endophyte then allows successful colonization by the endophyte, suggesting that survival of the endophyte in the intracellular environment is likely to be a specific adaptation^1,2,15^. For example, in the symbiotic interaction between *M. truncatula* and rhizobia, the *NAD1* (Nodules with Activated Defense 1) gene is expressed in the later stage of nodulation to maintain the rhizobial endosymbiosis in the nodule^55^, further studies are needed to determine the molecular mechanisms by which the plant allows *A. agilis* UMCV2 to thrive inside the plant.

The classification models of the metabolite profiles obtained by DLI-ESI-MS failed to discriminate between inoculated and uninoculated leaves at 5 d of the post-inoculation time; not even in m1 leaves in which a higher colonization was observed compared to m2 and m3 leaves. This result was not completely unexpected since one characteristic of commensal and mutualistic endophytes is that they do not cause any harm to the host plant, but under specific biotic or abiotic stresses, they may induce metabolic changes in the plant. Therefore, we conducted a finer study using GC-MS and observed an increased content of sugars in the inoculated m1 and m2 leaves compared to non-inoculated controls (*p* = 1.12 × 10^−5^). Sugar metabolism is essential for plant growth and development, but also it works as sensing elements to environmental stimuli, so alterations in the sugar content in plants have been reported as a response to biotic and abiotic signals^56–59^. It has been established that endophytes modulate the photosynthetic capacity of a plant which results in production of sugars that provide the basic carbon scaffolding necessary to form both plant and microbial structures^60^. We also noted that the sugar content did not increase in inoculated m3 leaves. The m3 leaf is the youngest leaf and is a sink tissue; thus, the presence of the endophyte would constitute an additional sugar demand.

In summary, our study showed that rhizobacteria *A. agilis* UMCV2 is able to colonize the inner leaf tissues of *M. truncatula* when it is inoculated in the foliage, but it is likely to be more easily established in the unifoliate leaves, so the hypothesis that the interconnected phenological age and chemical composition of the leaves affects the endophytic colonization is not discarded. These findings provide a first step in understanding the complexity of the metabolic environment that microorganisms must endure in the leaves following foliar fertilization. It will be very interesting to conduct a subsequent metabolomic studies to determine if *A. agilis* UMCV2 impacts or not in the broad range of compounds formed at early stages of the colonization or during the presence of biotic or abiotic stimulus, or furthermore, if *A. agilis* UMCV2 as a facultative endophyte contributes to the production of metabolites, that help the plant to adapt to, or cope with, environmental stimuli^61^.

## Materials and Methods

### Biological material and growth conditions

*Medicago truncatula* seeds (ecotype Jemalong A17) were chemically scarified^62^ with 1 mL of concentrated sulfuric acid; the seeds were immersed in a vial containing the acid, and then agitated for 8 min. The acid was decanted, and the seeds were rinsed with five washes of sterile deionized water. For sterilization, seeds were immersed in 12% sodium hypochlorite for  2 min and rinsed with abundant sterile deionized water. Seeds were placed on vertically oriented Petri dishes containing 1% agar and 0.6% sucrose and vernalized at 4 °C for 3 days. Seeds were germinated until the main root reached a length of 2 to 3 cm. One seedling was subsequently transferred into individual glass jars containing 20 mL of Hoagland medium and 1% agar. Later, the seedlings were returned to the Percival growth chamber with a photoperiod of 16 h a day at a light intensity of 200 μmol m^−2^ s^−1^ at 22 °C for 15 days.

The *M. truncatula* leaves were named according to a standardized numerical coding system^31^ to define the phenotypic development of this legume plant. The metamer associated with the unifoliate leaf was designed as metamer 1 (m1), and the units developing above as m2 (the first trifoliate leaf), and m3. Here, the 15-day-old plants collected for chemical and molecular analysis had the unifoliate leaf (m1) and the first and second trifoliate leaves (m2 and m3, respectively; Fig. 1a). The m1 emerged on the fourth day after seed germination; at harvest, therefore, m1 was at 11 days of development, m2 at 8 days, and m3 at 6 days.

The root-associated bacteria *A. agilis* UMCV2 was isolated from lightly acid soil, as previously described^29^. The bacterium was grown on nutrient agar (NA) at 26 °C.

### Inoculation experiments

A total of 72 plants from 10-day old were inoculated with an *A. agilis* UMCV2 suspension prepared in 0.2 M phosphate buffer (pH 7.4) until obtaining the optical density of 0.2 DO_590_. The other 72 uninoculated plants were brushstroked with phosphate buffer and used as controls.

The inoculation was made in one leaf per plant with a natural hair brush 3 mm in diameter. Two brushstrokes were made (approximately 2.2 × 10^6^ colony-forming units, CFU, per brushstroke), one in the upper surface and the other under the leaf, ensuring that none of the bacterial suspension fell into the growth medium. Plants were placed in three groups of twenty-four. In the first group of plants, only the m1 leaves were inoculated; in the second group, only the m2 were inoculated; and in the third, only the m3 were inoculated. The inoculated plants were returned to the Percival growth chamber, and the interaction was allowed to proceed for 5 days, until the plants were 15 days old. Then, the leaves were harvested for mass spectrometry analyses and qPCR bacterial DNA quantification.

To determine if a smaller bacterial concentration in the inoculum lead to a greater endophytic colonization, we performed an additional inoculation experiment, in which the m1 leaves were inoculated with a bacterial solution prepared at 0.1 DO_590_.

### Mass spectrometry analysis of leaves

Leaves (m1, m2, and m3) from 15-day-old control and inoculated plants were harvested, immediately frozen with liquid nitrogen, and individually stored at −80 °C until use. Then, the leaves were lyophilized and individually milled in a Mixer Mill (MM 400-Retsch, Verder Scientific GmbH & Co. KG; Haan, Germany) at 30 Hz for 20 s. The milled leaves were mixed with 0.5 mL of a cooled (4 °C) aqueous solution containing 75% methanol acidified with 0.1% formic acid, and later sonicated in an ultrasonic bath for 30 min^17^, and centrifuged at 15 000 rpm for 10 min at 4 °C. The extract was filtered through a 0.2-μm pore diameter filter for mass spectrometry analysis.

Each milled leaf was analyzed by direct liquid injection-electrospray ionization-mass spectrometry (DLI-ESI-MS)^63^ using a ZQ-detector 2 Waters® mass spectrometer in scan mode with a range of 50 to 2000 *m/z*, programmed with a cone temperature of 135 °C, capillary voltage of 3 V, and taking 1 scan s^−1^, until 1 minute of data acquisition. The aqueous solution containing 75% methanol acidified with 0.1% formic acid (blank sample) was injected at the beginning and at the end of each round of samples for washing purposes, and three technical samples were included at different days to corroborate reproducibility over time. The experiments were repeated three times.

### Statistical models for data analysis

For mass spectra processing, the *MALDIquant* package in R language was used^64^. The intensity of each ion signal (*m/z*) was normalized using the Statistics-sensitive Non-linear Iterative Peak-clipping (SNIP) algorithm to correct the baseline; and for ion detection, we used the absolute mean deviation (MAD) algorithm as the noise estimator with a signal-to-noise ratio of six. Finally, alignment of the detected signals was performed using a tolerance of ±0.35 *m/z*. We used a principal component analysis (PCA) to compare the DLI-ESI-MS metabolic fingerprint data of the leaves.

To determine the contribution of every ion, we used the *rattle* package (version 5.1.0) in R to construct a Random Forest (RF) model^65^. Implementation of the RF algorithm using *m/z* features, allows the creation of a large number of decision trees that classify the experimental units as belonging to any one treatment with high predictability and the characteristics that best predict it. For the model, 70% of the matrix was used to train the model, 15% for the validation step, and 15% for testing. Five hundred decision trees were built to finally obtain a predictive model that could classify samples depending on leaf age or the presence of the UMCV2 strain. Subsequently, the 30 most important ions for leaf classification were identified by the Mean Decrease Gini criterion; ions with higher values play the most important role in predicting the correct leaf samples classification. Analysis of ordination by PCA was performed again using the “classifier” ions. To validate the separation of the data obtained between treatments, a heat map was constructed in combination with a hierarchical cluster analysis using the Euclidean distance between experimental units and Ward’s algorithm for classification.

### Putative metabolite identification

The most important ions were putatively identified according to the RF algorithm, using SpiderMass software^66^ and a *M. truncatula* metabolite database (PlantCyc database, https://www.plantcyc.org/). Ion identification was based on the mass-to-charge fit with a tolerance of ±0.35 *m/z*.

### Analysis of soluble sugars

Sugars were extracted from 50 mg of controls and inoculated m1, m2, and m3 leaves with 1 mL of 80% ethanol at room temperature with continuous agitation for 12 h. Each treatment consisted of twenty composite samples of three plants. The sample was centrifuged at 15 000 rpm for 15 min and the extract was evaporated to complete dryness at 40 °C and then derivatized and analyzed using the GC-MS method according to a previous report^67^. The GC-MS analysis was performed in a gas chromatograph (Agilent 6850 Series II; Agilent, Foster City, CA, USA), equipped with an Agilent MS detector, model 5973, and an HP-5 MS capillary column (5% [w/v] phenyl methyl polysiloxane, 30 m × 0.25 mm I.D., film thickness of 0.25 μm). Glucose, fructose, myo-inositol, and sucrose were identified using a combination of a NIST 2.0 mass spectra database search and a comparison with the pure standard (Sigma-Aldrich) retention times and mass spectra. For quantification, the standards were mixed (1 μg each), derivatized, and 1 μL was injected into the GC. The peak area of each standard within the mixture was correlated with the peak area of the eluted compound in the sample. The analysis was repeated three times. Data were analyzed using a two factor ANOVA (inoculum and leaf) followed by a Tukey´s post-test (*p* ≤ 0.05).

### *In situ* hybridization assay

To corroborate that bacterial DNA detected by qPCR originated from bacteria exhibiting endophytic growth, inoculated leaves were randomly collected and images of the inner planes of the clarified tissues were obtained using a confocal microscope (FV1200, Olympus Corporation, Tokyo, Japan) as previously reported^20^. The inoculated bacteria were localized using fluorescence *in situ* hybridization, according to the FISH protocol described previously^68^. Actinobacteria 5 ‘Rhodamine Green TM-X (NHS Ester) TAT AGT TAC CAC CGC CGT was used^69^, which hybridizes with the 23 S ribosomal RNA of *A. agilis* and is visualized as green fluorescence (excitation and emission wavelengths: 502 and 527 nm, respectively).

### Bacterial quantification

Leaves (m1, m2, and m3) from control and inoculated plants (n = 8) were superficially disinfected with 20% sodium hypochlorite for 3 min, washed three times with abundant sterile deionized water, and ground in liquid nitrogen. The method for bacterial DNA quantification by qPCR was as previously described^20^. DNA was extracted from powdered leaves employing the methodology reported previously^70^; the 2CV2F and 2CV2R primers designed to specifically amplify an amplicon in the *A. agilis* UMCV2 16S–23S internal transcribed spacer (ITS) were used for absolute quantification of bacterial DNA in a StepOne thermocycler (Applied Biosystems, Foster City, CA, USA). A calibration curve was used from 100 ng to 20 fg with a coefficient of determination (*R*^*2*^) of 0.998 and a maximum dissociation peak at 83.78 °C. In each assay, 50 ng of total DNA was used as template. Three independent analysis were done. To estimate the number of CFUs in the inoculated leaves, we performed a regression curve by using the total viable account obtained from 100 μL of five serial bacterial dilutions (0.5, 0.4, 0.3, 0.2, and 0.1 at 590 nm) and the DNA content in each solution. Serial dilutions of each bacterial suspension were plated in nutrient agar and after 5 d of incubation at 23 °C, the colonies produced were counted to calculate the CFU at each bacterial suspension. The extraction of the total bacterial DNA was made from 1 mL of each bacterial suspension as previously described^70^. The amount of DNA was quantified with a Thermo Fisher NanoDrop 2000. The regressions curve produced with the data of total viable account and DNA amount of each bacterial suspension had a *R*^*2*^ value of 0.9448.

## Supplementary information

Supplementary Information

## Data Availability

All data generated in this study are available from the corresponding author, upon reasonable request.
