# Supplementary material for: Metabolomic effects of the colonization of Medicago truncatula by the facultative endophyte Arthrobacter agilis UMCV2 in a foliar inoculation system
Source: Sci Rep. 2020 May 21;10:8426. doi: 10.1038/s41598-020-65314-4 (PMC7242375; doi:10.1038/s41598-020-65314-4)
Supplement: Supplementary file 1 — Supplementary Information [file 41598_2020_65314_MOESM1_ESM.pdf]

Metabolomic effects of the colonization of *Medicago truncatula* by the facultative endophyte *Arthrobacter agilis* UMCV2 in a foliar inoculation system

Arturo Ramírez-Ordorica[1], Eduardo Valencia-Cantero[1], Idolina Flores-Cortez[1], María Teresa Carrillo-Rayas[2], Ma. Isabel Cristina Elizarraraz-Anaya[2], Josaphat Montero-Vargas[2], Robert Winkler[2], and Lourdes Macías-Rodríguez[1]*

[1]Instituto de Investigaciones Químico Biológicas, Universidad Michoacana de San Nicolás de Hidalgo, Edifico B3, Ciudad Universitaria, C. P. 58030, Morelia, Michoacán, México. [2]Department of Biotechnology and Biochemistry, Cinvestav Unidad Irapuato. Km 9.6 Libramiento Norte Carr. Irapuato-León. C. P. 36824, Irapuato, Guanajuato, México

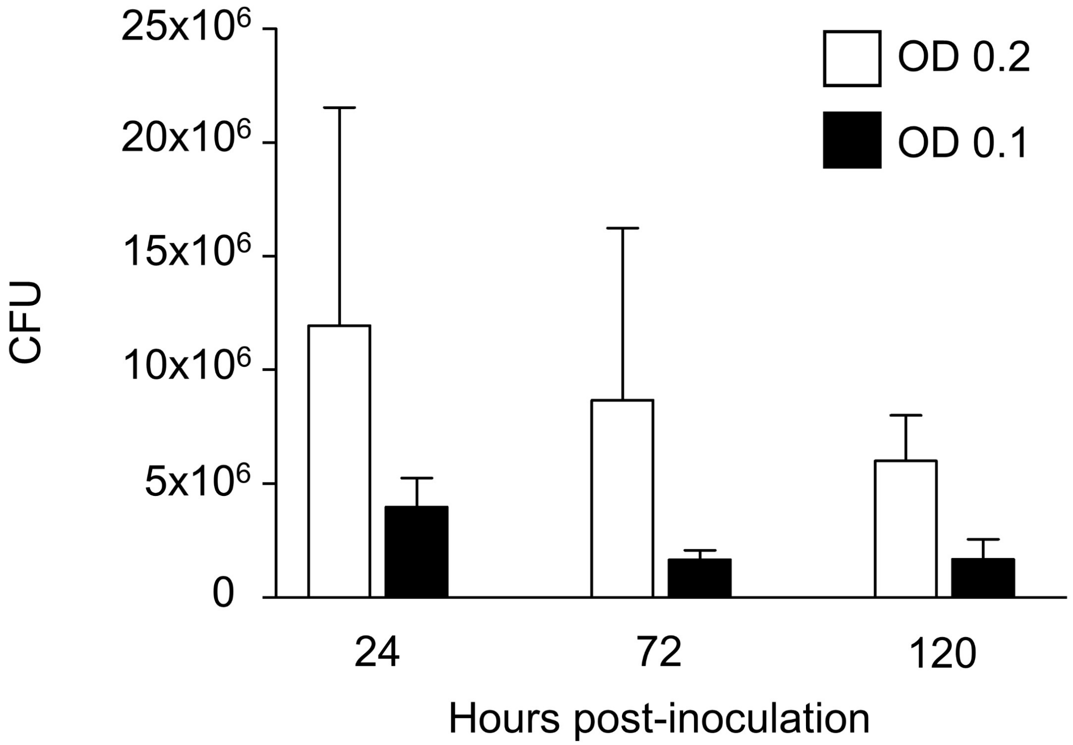

Supplementary Fig S1. Quantification of *A. agilis* UMCV2 in m1 leaves through the time at two different bacterial concentration in the inoculum. Bars represent the means ± standar error values (n=5). Tukey's post-test (α=0.05).
